# Supplementary material for: Are carbon emissions trading and green financial instruments synergistic? -Comprehensive quantitative research based on content analysis
Source: PLoS One. 2024 Mar 7;19(3):e0298601. doi: 10.1371/journal.pone.0298601 (PMC10919601; doi:10.1371/journal.pone.0298601)
Supplement: S1 Data — (ZIP) [file pone.0298601.s001.zip › Data/地级市Z_F工作报告.zip!/╔╜╬≈/╦╖╓▌╩╨/└┤╫╘╣1⁄2╓┌║┼í╛┬φ┐╦╩2╛▌═°í┐_2014.docx]

各位代表：

现在，我代表市人民政府向大会作工作报告，请予审议，并请市政协委员和其他列席人员提出意见。

一、2013年工作回顾

2013年，在省委、省政府和市委的正确领导下，我们深入贯彻党的十八大、十八届三中全会精神，紧紧围绕推进“两大任务”的工作主题，积极应对经济下行压力，统筹稳增长、调结构、促改革、惠民生，有效施策、攻坚克难，各项工作稳中有为、稳中有进，实现了全年经济社会发展主要预期目标，在全省目标责任考核中我市被省委省政府表彰为优秀市。

(一)积极应对经济下行压力，经济运行平稳健康。坚持稳中求进，采取了一系列改革发展措施。认真落实中央和省宏观调控政策，及时制定实施促进煤炭经济可持续增长、保障工业经济平稳健康发展等政策，扶持实体经济发展。扎实开展“朔州企业家投资在朔州”和“项目推进年”活动，省市两级重点工程完成投资905亿元。大力推进转型综改试验区建设，制定实施“十二五”后三年实施方案和2013年行动计划，强化科技引领，新建企业技术研发中心18家，申报专利1245件，总量跃居全省第三，增幅居全省第一;推进土地管理制度改革，开展采矿用地、工矿废弃地复垦等试点工作，保障建设用地需求;推进金融创新，促进银企合作，金融对地方经济的支持力度得到加强;加大招商引资力度，签约项目169个，到位资金740.8亿元，为经济发展增添了后劲。转型综改工作排名全省第一。在政策措施保障、实体经济支撑、项目投资拉动、改革创新驱动等多方面积极因素的共同作用下，我们赢得了稳步发展的局面。多项主要经济指标增幅位居全省前列。地区生产总值完成1026.4亿元，增长9%;工业增加值完成554.9亿元，增长11.5%;固定资产投资完成774.7亿元，增长26.9%;社会消费品零售总额完成218.8亿元，增长14.5%;公共财政预算收入完成95.3亿元，增长13.1%;城镇居民人均可支配收入达到24013元，增长9.9%;农民人均纯收入达到9040元，增长13%;居民消费价格涨幅2.9%。

(二)加快调整产业结构，发展质量得到提升。大力改造提升传统产业，制定实施加快推进煤电一体化、促进工业经济循环发展等意见。加快建设现代化矿井，标准化矿井达到35座，原煤产量达到2.2亿吨。壮大电力产业，全市电力装机容量达到754.95万千瓦，发电量完成278.7亿度，其中风力发电、太阳能发电、生物质能发电等新能源电力装机规模达到121.5万千瓦，居全省第一。促进陶瓷产业提档升级，日用瓷产量达到16.2亿件，居全省第一。稳定发展乳品产业，乳制品产量完成28.1万吨，居全省第一。加快发展循环经济，积极扶持循环经济企业和高端项目，粉煤灰综合利用企业达到50家，年消化粉煤灰330万吨，居全省第一。亚洲粉煤灰协会年会在我市召开，具有朔州特色的循环经济产生国际影响。培育壮大新兴产业，铺开装备制造、新型材料、医药、化工、特色食品加工等新兴产业项目99项，总投资达726.4亿元，其中22个投资5亿元以上项目完成投资118亿元。非煤电工业完成投资145.5亿元，增长56.3%。服务业完成投资365.3亿元，增长22.3%。第三产业投资额超过第二产业投资额。旅游总收入达到84.1亿元，增长29.8%。产业结构发生积极变化，一二三产占地区生产总值比重调整为6：56：38。

(三)加大“三农”工作力度，生态畜牧经济区建设成效显著。大力实施强农惠农富农政策，各级财政投入“三农”资金达20亿元。加强农田水利基本建设，农田实灌面积达到198万亩。粮食总产量达到23.5亿斤，再创历史新高。加快发展特色农业，新增110个“一村一品”专业村，全市省级示范村达到317个，六县区全部进入全省30个牛羊产业重点县行列。农产品加工龙头企业销售收入达到130亿元。大力推进产业扶贫开发、干部包村增收和机关定点扶贫，又有1.6万贫困人口实现脱贫。扎实推进“五件实事”，改造农村困难家庭危房14800户，易地搬迁特困群众1596户，改扩建村级幼儿园20所，乡村清洁工程全面启动，行政村街道亮化任务在全省率先完成。同时，为全市农户免费发放43万吨取暖煤;财政投资7355万元，集中解决了88个农村的饮水困难。我市被省委省政府表彰为全省粮食生产先进市、全省增加农民收入先进市、全省行政村主街道路灯亮化工作先进市、全省百企千村产业扶贫先进市。

(四)大力推进城市建设，城镇化水平稳步提高。按照“规划引领、完善功能、提升品位、文化塑造、生态支撑”的理念，深化“五城联创”，全面推进国家园林城市、国家卫生城市、国家环保模范城市、国家双拥模范城市、全国文明城市创建活动。加大中心市区建设力度，改扩建7条城市道路，新增绿地面积21.56万平方米，建成区绿化覆盖率达到42.02%;拆除小锅炉20座，增加集中供热面积3.04万平方米;特别是下决心启动了市民期盼已久的七里河综合治理工程，城市面貌加快改变。积极推进大县城和重点镇建设。全市城镇化率达到51.09%，比上年提高1.1个百分点。加强公路建设，完成投资24.35亿元，通车里程突破1万公里。建成朔州环城高速西南段、荣乌高速公路山阴至平鲁段以及贯穿平川四县区的旅游生态观光路。持续推进植树造林，投入12.8亿元，营造林33万亩。积极推进节能减排，万元地区生产总值综合能耗下降3.64%，主要污染物减排任务全部完成。我市荣获国家园林城市称号。

(五)着力保障和改善民生，社会事业全面进步。始终把改善民生作为工作的出发点和落脚点，在财政增收难度加大的情况下，财政支出进一步向民生倾斜，全市财政在民生领域投入124.65亿元，占一般预算支出的80.7%。加快发展教育。新改扩建32所公办幼儿园。高考二本以上达线率达到34.61%，高出全省平均水平9.87个百分点。中北大学朔州校区招生规模进一步扩大，朔州师专正式挂牌。积极发展医疗卫生事业。深化医药卫生体制改革，积极推进县区公立医院改革。县乡村三级医疗卫生机构达标率达到100%。计划生育工作排名同类市全省第一。大力发展文化事业。实施送戏下乡等文化惠民工程，启动创建第二批国家公共文化服务体系示范区。全民健身活动蓬勃开展，竞技体育实现突破，玉龙马术队代表山西参加第十二届全运会。稳定扩大就业。新增城镇就业人数2.4万人，完成省下达任务的111%。着力增加居民收入。提高企业工资指导线、最低工资标准、机关事业单位人员津补贴和取暖补贴标准，落实带薪年休假制度。加强社会保障体系建设。提高城乡居民低保标准、新农合财政补助标准、企业退休人员基本养老金、城乡居民基础养老金以及20种重大疾病补偿比例，制定实施城乡居民临时救助办法和农村五保户供养办法。继续推进保障性安居工程建设。新开工城镇保障性住房19125套，完成农村住房抗震改建3000户。高度重视安全生产工作。扎实开展安全生产大检查和“回头看”活动，生产经营性事故死亡人数下降36.5%，占省定控制指标的69.64%，煤矿百万吨死亡率为零。我市被表彰为治超工作优秀市。深入开展平安朔州建设。健全社会治安防控体系，着力做好信访工作，积极排查化解社会矛盾，社会保持和谐稳定。我市被中央有关部门表彰为全国社会管理综合治理优秀市。与此同时，加强国防后备力量建设，积极发展妇女儿童、老龄和残疾人事业，民族、宗教、地震、气象、应急、档案、地方志等各方面工作都取得新成绩。

我们自觉接受人大和政协监督，全年共办理人大代表建议和政协委员提案181件。深入推进“六五”普法。大力推进行政审批制度改革，市政府各部门取消行政审批事项47项，下放12项,保留的99项审批项目全部进入政务服务中心，全部纳入电子监察系统。严格执行中央八项规定和省市有关规定，坚决反对“四风”，全市会议费下降49.97%，“三公”经费下降12.83%。大力开展“右玉精神在朔州”和“看作为、比建树、争一流”活动，干部作风明显改进，改革创新和干事创业氛围更加浓厚。各位代表，2013年是我们站在新的起点上完善新思路、推出新举措、实现新发展的一年，成绩来之不易。这是省委、省政府和市委正确领导的结果，是市人大、市政协支持监督的结果，是全市人民共同努力的结果。在此，我代表市人民政府，向全市人民，向驻朔部队、武警官兵，向中央和省属驻朔单位，向民主党派、工商联、无党派人士、人民团体，向各位人大代表、政协委员，向所有关心支持朔州改革发展的朋友们，表示崇高的敬意和衷心的感谢!

在肯定成绩的同时，也要清醒地看到，我市经济社会发展中还存在不少困难和问题。主要是：受宏观经济下行压力影响和一煤独大结构制约，稳中求进难度很大;支撑“两大任务”的大项目、好项目不多，创新驱动能力不强;农业基础薄弱，农民持续增收困难较大;民生社会事业和城市基础设施建设滞后，公共服务能力不足;安全生产基础不牢，节能减排和环境保护任务艰巨;一些公务员思想观念陈旧僵化，专业水平不高，执行力不强，违规违纪、腐败问题时有发生;政府职能转变相对滞后，群众不满意的问题较多，政务环境需要进一步优化。

我们是人民政府，必须秉持“意莫高于爱民，行莫厚于乐民”的执政理念和服务意识。我们将牢记使命，正视问题，直面挑战，勤勉工作，廉洁从政，努力在新的一年向朔州人民交上一份满意答卷。

二、2014年工作安排

2014年是贯彻落实党的十八大、十八届三中全会精神，全面深化改革的一年，是扎实推进“两大任务”、加快朔州转型跨越发展的关键之年。

当前，我们面临的形势依然错综复杂，但仍处于可以大有作为的重要战略机遇期。中央推进全面深化改革，改革红利、发展潜力、市场活力将得到进一步释放。我省转型综改区建设向纵深推进，我市的发展环境会更为有利。我们必须牢牢抓住机遇，坚定不移推进“两大任务”，敢于担当责任，勇于直面矛盾，善于破解难题，为朔州发展做更多实事，为朔州人民谋更多福祉。

今年政府工作的总体要求是：高举中国特色社会主义伟大旗帜，以邓小平理论、“三个代表”重要思想、科学发展观为指导，全面贯彻落实党的十八大、十八届三中全会和习近平同志系列重要讲话精神，紧扣“两大任务”，坚持稳中求进，勇于改革创新，着力做好煤电一体发展、循环经济发展、现代农业发展“三篇文章”，着力打造综合能源、固废利用、日用陶瓷、畜牧养殖、特色农产品加工“五个基地”，着力保障和改善民生，深入开展党的群众路线教育实践活动，全面推进经济、政治、文化、社会和生态文明建设，促进经济持续健康发展，保持社会和谐稳定，为推进转型跨越发展，建设美丽朔州、塞上明珠而努力奋斗。

今年经济社会发展的主要预期目标是：地区生产总值增长10%左右，工业增加值增长12%，固定资产投资增长23%，社会消费品零售总额增长14%，公共财政预算收入增长11%，城镇居民人均可支配收入增长11%，农民人均纯收入增长12%，城镇新增就业岗位2.4万个，居民消费价格总水平涨幅控制在3.5%左右。约束性指标是：万元地区生产总值综合能耗下降3.5%，二氧化硫、化学需氧量、氨氮、氮氧化物、烟尘、粉尘减排完成省下达任务，万元工业增加值用水量下降3.2%。主要工作任务是：

(一)巩固煤电，壮大多元，加快促进经济提质增效升级。

以深化改革为动力，以优化结构为主攻方向，围绕巩固煤电、壮大多元，认真实施转型综改2014年“223”行动计划，着力抓好重大改革、重大事项、重大项目，深入推进综改攻坚、创新驱动、项目见效，加快破解制约资源型经济转型发展的体制机制障碍和要素瓶颈，在转变发展方式中实现稳中有进。

推进煤电一体化发展。落实煤炭探矿权、采矿权改革措施，加快建设现代化矿井，合理安排煤炭新增产能，主动防范产能过剩风险，促进煤炭工业可持续发展。抓住国家实施以电代煤、以电代油电能替代工程机遇，推动电力工业高效清洁发展。积极发展低热值煤发电和风能、太阳能、生物质能等新能源电力产业，加快建设电力外送通道。完善煤电一体化机制，促进煤电联营发展，提高煤炭就地转化率。年内新增5—10座标准化矿井，推动平朔2×66万千瓦、平朔2×35万千瓦、同煤朔南2×35万千瓦、山西国际能源昱光二期2×35万千瓦等低热值煤发电项目和中电国际神头发电公司2×100万千瓦项目、16个风电项目开工建设。

实施重大项目引领带动战略。坚持“六位一体”推进重点工程建设，扎实开展“项目见效年”活动，充分发挥投资拉动和大项目带动作用。以政府投资为引导，多渠道筹措建设资金，加强基础设施、产业转型、生态环保和民生事业等重点领域投资。实施省市重点工程266项，当年完成投资658亿元。集中力量，主攻十大重点工程：高速铁路、高速公路、飞机场为重点的重大交通设施工程，七里河综合治理工程，电力建设工程，朔城区北京电子城，平鲁区光电信息产业园，山阴县大型煤化工项目，怀仁县金沙滩陶瓷建材项目群，应县恒天然奶牛牧场群，右玉县食品工业项目群，开发区钒电池产业化项目。十大重点工程事关长远发展，事关民生福祉，要加强组织领导，统筹协同推进，务求取得实效。

大力发展循环经济。坚持把循环经济作为朔州转型发展的一条基本路径。设立循环经济发展专项资金，市财政出资3000万元，各县区和开发区分别配套1000万元，形成1亿元的资金规模。加快推进粉煤灰资源化利用，重点抓好固废园区建设。积极推动晋北煤化工基地建设。启动实施国家创新驱动发展战略朔州行动计划和低碳创新行动计划，深化与北京大学、中北大学等高等院校和科研单位的产学研合作，引进百名紧缺人才，支持企业技术研发，加强以煤基科技、低碳科技为主攻方向的科技攻关，打牢建设全国工业固废综合利用示范基地和新型煤化工基地的项目和技术基础。

积极培育新兴产业。坚持把新兴产业作为转型发展的着力点。深入开展“朔州企业家投资在朔州”活动，鼓励驻朔企业和民营企业投资新兴产业，培育壮大装备制造和新能源汽车产业，提升陶瓷产业，做大特色农产品加工业，加快发展高新技术产业。各县区、开发区今年至少要开工建设3个投资5亿元以上的产业转型项目，进一步夯实转型发展基础。

加快发展服务业。落实增加居民收入的各项政策，提高消费能力，促进服务业发展。鼓励发展电子商务，规划建设城市综合体，打造特色商业街，完善农产品流通体系，扩大日用瓷出口，提升商贸流通业发展水平。依托丰富的历史文化资源和独特的自然生态风光，加快发展文化旅游业。开展“美丽朔州清凉游”活动。加强景区建设，重点推进雁门关大广武旅游区项目，建设集文物保护开发、边塞风情体验、影视制作为一体的文化旅游景区。

提高开发区和工业园区发展水平。开发区和工业园区是转型发展的主要载体，要完善基础设施，明确主打产业，构建产业链条，理顺体制机制，优化服务环境，加快上档升级。要以工业园区为主体，扩大对外开放，狠抓招商引资，对接京津冀，融入环渤海，承接大产业，落地好项目。八大工业园区今年至少要开工建设两个投资5亿元以上的产业转型项目。力争有三个园区建成省级开发区。

支持企业健康发展。积极落实扶持企业的各类政策，进一步研究制定政策措施，减轻企业负担，促进企业发展。创新金融服务，推进银企对接，培育资本市场。组建朔州农村商业银行，完善金融体系，强化金融对地方经济的支持。健全企业服务体系，加大对中小企业产品开发、品牌创建、市场开拓的支持力度，培育更多年销售收入超亿元的“小巨人”企业。(二)以农载牧，以牧富民，加快推进农业现代化。

始终把做好“三农”工作作为全部工作的重中之重，以促进农民增收为核心，以“一村一品、一县一业”为抓手，以农民户均1个大棚、1头牛、人均10只羊的“1110”为目标，坚持以农载牧、以牧富民，加快发展特色现代农业，当好雁门关生态畜牧经济区建设排头兵。

强化农业支持保护政策。在落实好中央和省各项强农惠农富农政策的基础上，进一步加大财政对农业生产的补贴和奖励补助力度，重点扶持规模种植、规模养殖和特色农畜产品加工项目。设立现代农业专项扶持资金，市财政出资3000万元，各县区分别配套1000万元，形成9000万元的资金规模。统筹整合涉农资金，提高资金使用效益。不管财力多么紧张，都要确保农业投入只增不减。

大力发展特色现代农业。深入推进“一村一品、一县一业”，大力实施农民户均增收万元工程。今年新增省级“一村一品”建设示范村110个，进一步提升六个基地县发展水平。实施百万亩玉米高产创建地膜覆盖工程，打造一批吨粮示范田。实施杂粮产业振兴计划，建设有机燕麦、有机荞麦、优质马铃薯种植基地。实施高效设施农业工程，发展集中连片设施农业示范园区，扩大设施蔬菜种植面积。实施规模健康养殖工程，建设一批现代化养殖园区和大型养殖牧场。加快推进应县恒天然奶牛牧场群和山阴县10个千头现代化牛场。加强农田水利基本建设，增强农业综合生产能力。

加快构建新型农业经营体系。深化农村改革试验区建设，做好土地承包经营权确权登记颁证工作，引导农村土地有序流转，培育专业大户、家庭农场、农民合作社、农业企业等新型农业经营主体，发展多种形式适度规模经营。培育壮大农产品加工龙头企业，支持古城集团、梨花春集团、怀仁金沙滩羔羊肉业公司、右玉臣丰食业公司等企业做大做强，发展订单农业，推广公司加基地、基地连农户的经营模式，提高农业产业化发展水平。鼓励农产品加工企业整合重组，加强品牌建设，提高市场竞争力。推进农业科技创新，加快发展农业机械化。推进供销合作社改革发展，健全农业社会化服务体系。

深入实施产业扶贫开发工程。创新扶贫方式，整合扶贫资源，实施精准扶贫。积极推进中煤华昱能源公司现代农业示范园区、同煤集团鑫邦燕麦5万吨燕麦加工、阳煤集团晋西北1万吨优质牛羊肉加工等产业扶贫项目，促进农民增收和企业转型。鼓励引导社会力量参与扶贫事业，继续开展领导干部包村增收、机关定点扶贫工作。今年再实现2万贫困人口脱贫。

着力改善农村人居环境。坚持巩固两轮“五个全覆盖”成果与办好“五件实事”相结合，因地制宜，分类指导，大力实施以农村基础设施和公共服务为重点的完善提质工程，以采煤沉陷区治理、易地搬迁、危房改造为重点的农民安居工程，以垃圾污水治理为重点的环境整治工程，以美丽乡村建设为重点的宜居示范工程，加大中心村建设力度，逐步改善农村生产生活条件。

继续办好“五件实事”。今年再改造农村困难家庭危房7100户，改造农村幼儿园14所，易地搬迁农村贫困人口2238人，深入推进乡村清洁工程，在已完成行政村街道亮化工程的基础上培训1万名新型职业农民。

(三)完善功能，提升品位，加快建设塞上明珠。

坚持以人为本、四化同步、优化布局、生态文明、文化传承的原则，以“五城联创”为载体，加快扩容提质，积极推进特色新型城镇化，努力建设彰显自然生态精致宜居现代化城市魅力的塞上明珠。

加快提高中心城市建设和管理水平。根据建设晋北南部区域性中心城市的功能定位，按照“一山两河一湖”的总体布局，大力加强基础设施建设，完善公共服务体系，不断增强中心城市的综合承载能力和辐射带动能力。着眼于解决城市发展面临的突出问题，下力气推进十大城建工程。一是规划修编工程。创新规划理念，完成总规修编，完善规划程序，强化规划管控，健全规划管理体制机制，提高城市规划管理水平。二是七里河综合治理工程。通过城中村拆迁、河道治理、绿化美化，把七里河建设成为朔州的标志性景观。三是朔州老城改造完善工程。实施城墙修复，完善老城内部设施，留住朔州历史记忆、文化脉络、地域风貌。四是路桥建设工程。修通开发路恢河大桥，改扩建部分城市主街道，完善路网结构，提高城市的通达性。五是供水工程。改造提升现有三个自来水厂，建设第四自来水厂。六是集中供热工程。取缔燃煤小锅炉，改造城中村，整合供热企业，推进热电联产集中供热全覆盖。七是气化工程。拓展市区燃气管网，建设天然气液化工厂和加气站，提高燃气供应率。八是净化工程。改造市污水处理厂，建设市第二污水处理厂，完成垃圾处理城南填埋区异地建设工程，增建城市公厕，提高城市污水处理率和垃圾无害化处理率。九是绿化工程。完善绿地规划，加强绿地管护，提升城市绿化水平。十是优质公交工程。整合重组市公交公司，规划建设公交运营设施，更新公交汽车，整顿规范出租车营运市场秩序，提高公共交通服务能力。城市建设是一项综合工程，是一场社会变革，要秉承多留遗产、少留遗憾的理念，坚持高标准规划、高起点设计、高水平建设、精细化管理，以创造历史、追求艺术的高度负责精神，努力打造城市建设的精品力作，提高城市可持续发展能力，真正实现城市让生活更美好。

推进县域城镇化。加快推进以大县城和中心镇为重点的县域城镇化，推动城镇化、工业化、农业现代化协调发展，促进城镇发展同产业支撑、就业转移、人口集聚相统一，带动县域经济发展壮大。平鲁区要加快与中心市区同城化步伐，早日实现“两个70%”。山阴县要加大产业转型力度，建设生态新城。怀仁县要抢抓撤县设市机遇，进一步提高城镇化水平。应县要建设旅游文化名城，做大经济总量。右玉县要发挥生态优势，打造生态旅游胜地。坚持城乡统筹发展，推进城乡规划、基础设施和公共服务一体化。加强交通体系建设，完善高速公路网，改造提升乡村公路，发挥交通网络对城镇化的支撑和引导作用。

继续推进“五城联创”。巩固国家园林城市创建成果，引深创建活动，进一步提升园林城市水平。统筹推进国家卫生城市和国家环保模范城市创建工作，争取早日申报。积极推进国家双拥模范城市创建工作，广泛开展军民共建活动，支持国防和军队建设，推动军民融合深度发展，力争今年进入省级双拥模范城市行列。扎实推进省级文明城市创建活动，为创建全国文明城市打好基础。

建立健全城镇化体制机制。加快建立规范透明的城市建设投融资机制，加强政府融资平台建设，通过多种渠道吸引社会资本参与城市建设。继续推进户籍制度改革，促进农业转移人口市民化。深化土地管理制度改革，积极推进重度盐碱地转为建设用地试点工作，进一步推进城乡建设用地增减挂钩等改革。坚持节约集约利用土地，强化建设用地效益考核。健全城镇住房制度，加快构建以政府为主提供基本保障、以市场为主满足多层次需求的住房供应体系。落实调控政策，促进房地产市场健康发展。

加强生态文明建设。把生态文明理念融入城镇化进程，强化环境保护和生态修复。坚持生态效益与经济效益兼顾，因地制宜，合理规划，持续推进造林绿化。实施好京津风沙源治理工程、三北防护林工程、退耕还林巩固成果工程和天然林保护工程，加强洪涛山防护林带、西北部百万亩樟子松基地、南山坡经济林区等区域林业生态建设，加大荒山荒坡绿化力度，搞好市区环城高速公路和荣乌高速公路绿化工程，全年营造林33万亩。加大森林资源管护力度，巩固绿化成果。加强水生态建设，推进桑干河、恢河、神头泉、太平湖综合治理，新建应县小石口水库、怀仁县东作里水库。加强饮用水源保护，确保饮水安全。狠抓大气污染防治和节能降耗，深入开展重点行业脱硫、脱硝、除尘改造，搬迁城市建成区范围内工业污染企业，淘汰市区建成区范围10蒸吨以下工业燃煤锅炉，淘汰黄标车，强化扬尘治理。完善生态补偿机制，推进采空区、沉陷区、水土流失区的生态治理修复。严肃查处环境违法行为。(四)改善民生，优化服务，加快提高人民生活水平。

提供优质公共服务，维护社会公平正义，增进人民福祉，是政府根本职责所在。要按照建机制、补短板、兜底线的要求，以更大的投入和更有力的举措，深化社会体制改革，加快健全基本公共服务体系，努力让人民过上更好生活。

优先发展教育。提高义务教育阶段学生生均公用经费标准，优化学校布局，促进义务教育均衡发展。加强市区中小学校建设，新建市一中高中部。推进幼儿园建设，市区5万平方米以上新建住宅小区都要配套建设幼儿园。加快发展职业教育，实施特殊教育提升计划。积极发展高等教育，完成中北大学朔州校区一期工程建设和基础实验室配套，提升朔州师专和朔州职业技术学院办学水平。加强教师队伍和师德建设，切实减轻学生课业负担，促进学生健康成长。

大力发展医疗卫生事业。完善基本药物制度，健全全民医保体系，提高城乡居民基本医保财政补贴标准，完善重特大疾病医疗保障和救助制度。提升乡村卫生院(室)医疗水平，加强社区卫生机构建设，做好市人民医院升级为三级综合医院的配套保障工作，筹建朔州大医院。加强医德医风建设，提升医疗服务质量。做好人口计生工作，落实一方是独生子女的夫妇可生育两个孩子的政策，实施计划生育失独家庭养老扶助。

加快文化改革发展。积极创建国家公共文化服务体系示范区。完善公共文化服务设施网络，建好管好用好图书馆(农家书屋)、文化馆(乡镇文化站)、科技馆、博物馆。建立完善公共文化服务保障机制，扩大政府文化资助和文化采购。加强文化遗产保护。广泛开展全民健身活动，举办朔州市第二届全民运动会。深化文化体制改革，政府部门要由办文化向管文化转变，整合市属文化企业，推动文化事业单位组建理事会。

积极扩大就业。推进以创业孵化基地为载体的国家级创业型城市建设，实施大学生创业引领计划和就业促进计划，政府购买基层公共管理和社会服务岗位更多用于吸纳高校毕业生就业，统筹做好农村转移劳动力、城镇困难人员、退役军人等就业工作。

完善社会保障体系。推进社会保险参保全覆盖，推广社会保障卡应用，加大五险统征力度，杜绝因拖欠社会保险而引起的群体性上访，构建和谐稳定的劳动关系。整合城乡居民基本养老保险制度，提高企业退休人员基本养老金和城乡低保水平，筹建市级老年公寓。稳定物价水平，完善社会救助和保障标准与物价上涨挂钩的联动机制。建立以财政投入为主的临时救助基金，为特殊困难群众基本生活提供保障。健全应急保障机制，建立市级应急物资储备制度，建设应急救灾物资储备库。新建各类保障性住房1.27万套。保障妇女儿童权益，做好残疾人基本公共服务，支持慈善事业发展。

坚持不懈抓好安全生产。安全生产是红线、是高压线，任何人任何时候都不能碰。严格实行网格化监管，强化属地管理、行业管理，构建“定格、定点、定人、定责、定制”的网格化管理体系，把政府安全生产监管责任落实到位。继续推进煤矿、非煤矿山、交通运输、危险化学品等重点行业企业安全生产标准化建设，落实企业安全生产主体责任。广泛开展安全乡村、安全社区创建活动，全面构建安全防控体系。按照“全覆盖、零容忍、严执法、重实效”的要求，持续开展以煤矿为重点覆盖各行业各领域的安全隐患排查治理，出重拳“打非治违”。严肃追究责任，严格落实安全生产目标责任考核“一票否决制”，坚决遏制重特大事故，减少一般性事故。加强安全生产教育培训，提高全社会的安全意识和防范能力。

推进社会治理创新。加强基层社会管理和服务体系建设，增强城乡社区服务功能，引导社会组织健康有序发展。搞好第三次全国经济普查。健全和落实重大决策社会稳定风险评估机制。改革信访工作制度，及时就地化解社会矛盾。深入开展普法教育，提高全社会法治意识。加强社会治安综合治理，完善防控体系，严厉打击各类违法犯罪行为，扎实推进平安朔州建设。

朔州人民对美好生活的向往就是我们的努力方向。着眼于解决人民群众最关心最直接最现实的利益问题，今年市政府要为民办好十件实事。一是新改扩建公办标准化幼儿园12所。二是实施学生饮用奶全覆盖工程。免费范围由义务教育阶段寄宿学生逐步扩大到全市义务教育阶段所有学生。三是促进高校毕业生就业。采取政府购买服务的方式，在乡镇农技推广、基层文化管理、食品药品监管、公共卫生服务等方面，为高校毕业生提供1000个就业岗位、组织3000名高校毕业生参加就业见习。四是提高城乡居民基础养老金标准。并轨实施朔州市农村老年人生活补助与城乡居民基础养老金两项制度，合并发放城乡居民基础养老金，年龄统一为60周岁，发放标准提高到每人每月95元。五是提高农村五保户供养标准。平均每人每年由目前的分散供养2119元和集中供养2802元，分别提高到2708元和3791元以上。六是实行异地就医即时结算报销。实现太原、大同两个我市转外就医最集中地区的联网结算。七是提供玉米、奶牛、森林等农业政策性保险。八是实施饮水解困工程。今年解决24个村8300人的饮水困难。九是建设100个副食品生产基地惠民直销店。十是送戏下乡。给常住人口在300人以上的行政村每年送戏3场。

民生无小事，枝叶总关情。我们要更加关注民生，切实保障民生，不断改善民生，把关系民生的工作一项一项抓紧抓好，一件一件落到实处，让广大人民群众不断享受到越来越多的改革发展成果，生活越来越幸福!

(五)深化改革，转变职能，加快建设法治政府和服务型政府。

改革是今年政府工作的首要任务。要抓住理顺政府与市场关系这个核心，深化行政体制改革，创新行政管理方式，加快转变政府职能，增强政府执行力和公信力。

加快转变职能。进一步简政放权，是政府的自我革命。深化行政审批制度改革，承接好上级下放的行政审批事项，继续取消和下放市级行政审批项目，公布市级政府行政审批清单。深化投资体制改革，落实企业投资自主权，使投资创业更加便利高效。深化工商登记制度改革，落实认缴登记、先照后证、年报公示等制度。完成市县政府机构改革，推进事业单位分类改革。各级政府要切实履行好公共服务、市场监管、社会管理、环境保护等职责，加大政府购买公共服务力度，为人民群众提供优质高效服务。

加强事中事后监管。坚持放管并重，建立纵横联动协同管理机制，实现责任和权力同步下放、放活和监管同步到位。加强政务服务中心管理，优化流程，提高效率。大力整顿和规范市场秩序，严厉打击制售假冒伪劣等侵害消费者权益的行为。健全食品药品安全监管体制，建立从生产加工到流通消费的全过程监管机制和可追溯体系，保障人民群众饮食用药安全。建设社会信用体系，褒扬诚信，惩戒失信。

强化权力运行制约和监督。坚持用制度管权管钱管事管人，把权力关进制度的笼子，让权力在阳光下运行。建立健全工程建设项目招投标、政府采购、国有资产管理等制度。严格财政资金管理和监督，把所有收支纳入预算管理，政府预算和决算向社会公开，部门预算要逐步公开到基本支出和项目支出，所有财政拨款的“三公”经费都要公开，所有公共资金、公共资产、公共资源都要进行审计，都要接受社会监督。要集中财力办大事，办群众期盼的事。不能乱花一分公款，不能私用任何权力。深入推进反腐倡廉制度建设，以铁的意志反对腐败，坚决查处违法违纪案件，切实做到干部清正、政府清廉。

各位代表，为政皆在民事，功业全凭实干。我们将把群众满意作为政府工作的第一标准，坚持问计于民、问政于民、问需于民，求真务实，真抓实干，努力做出实实在在的工作业绩。认真开展党的群众路线教育实践活动，严格执行中央八项规定，加大“四风”治理力度，切实解决门难进、脸难看、事难办等群众反映强烈的问题。深入开展“右玉精神在朔州”和“看作为、比建树、争一流”活动，不断改进工作作风。所有公务员特别是各级领导干部要始终坚持为民务实清廉，严以修身、严以用权、严以律己，谋事要实、创业要实、做人要实，当好人民公仆。

我们将自觉接受人大及其常委会监督，虚心接受人民政协的民主监督，认真听取人大代表、政协委员、民主党派、工商联、无党派人士和各人民团体的意见，不断增强依法治市、依法行政、依法办事的自觉性。

各位代表，全面深化改革的号角已经吹响，转型跨越发展的信心更加坚定。让我们紧密团结在以习近平同志为总书记的党中央周围，在省委、省政府和中共朔州市委的坚强领导下，担当起使命和责任，履行好职责和义务，为朔州人民的美好生活，为朔州的改革发展而努力奋斗!
